# ViMRT: a text-mining tool and search engine for automated virus mutation recognition

**DOI:** 10.1093/bioinformatics/btac721

**Published:** 2022-11-07

**Authors:** Yuantao Tong, Fanglin Tan, Honglian Huang, Zeyu Zhang, Hui Zong, Yujia Xie, Danqi Huang, Shiyang Cheng, Ziyi Wei, Meng Fang, M James C Crabbe, Ying Wang, Xiaoyan Zhang

**Affiliations:** Research Center for Translational Medicine, Shanghai East Hospital, School of Life Sciences and Technology, Tongji University, Shanghai 200092, China; Research Center for Translational Medicine, Shanghai East Hospital, School of Life Sciences and Technology, Tongji University, Shanghai 200092, China; Research Center for Translational Medicine, Shanghai East Hospital, School of Life Sciences and Technology, Tongji University, Shanghai 200092, China; Research Center for Translational Medicine, Shanghai East Hospital, School of Life Sciences and Technology, Tongji University, Shanghai 200092, China; Research Center for Translational Medicine, Shanghai East Hospital, School of Life Sciences and Technology, Tongji University, Shanghai 200092, China; Research Center for Translational Medicine, Shanghai East Hospital, School of Life Sciences and Technology, Tongji University, Shanghai 200092, China; Research Center for Translational Medicine, Shanghai East Hospital, School of Life Sciences and Technology, Tongji University, Shanghai 200092, China; Research Center for Translational Medicine, Shanghai East Hospital, School of Life Sciences and Technology, Tongji University, Shanghai 200092, China; Research Center for Translational Medicine, Shanghai East Hospital, School of Life Sciences and Technology, Tongji University, Shanghai 200092, China; Department of Laboratory Medicine, Shanghai Eastern Hepatobiliary Surgery Hospital, Shanghai 200438, China; Wolfson College, Oxford University, Oxford OX2 6UD, UK; Institute of Biomedical and Environmental Science & Technology, University of Bedfordshire, Luton LU1 3JU, UK; School of Life Sciences, Shanxi University, Taiyuan 030006, China; Research Center for Translational Medicine, Shanghai East Hospital, School of Life Sciences and Technology, Tongji University, Shanghai 200092, China; Department of Laboratory Medicine, Shanghai Eastern Hepatobiliary Surgery Hospital, Shanghai 200438, China; Research Center for Translational Medicine, Shanghai East Hospital, School of Life Sciences and Technology, Tongji University, Shanghai 200092, China

## Abstract

**Motivation:**

Virus mutation is one of the most important research issues which plays a critical role in disease progression and has prompted substantial scientific publications. Mutation extraction from published literature has become an increasingly important task, benefiting many downstream applications such as vaccine design and drug usage. However, most existing approaches have low performances in extracting virus mutation due to both lack of precise virus mutation information and their development based on human gene mutations.

**Results:**

We developed ViMRT, a text-mining tool and search engine for automated virus mutation recognition using natural language processing. ViMRT mainly developed 8 optimized rules and 12 regular expressions based on a development dataset comprising 830 papers of 5 human severe disease-related viruses. It achieved higher performance than other tools in a test dataset (1662 papers, 99.17% in F1-score) and has been applied well to two other viruses, influenza virus and severe acute respiratory syndrome coronavirus-2 (212 papers, 96.99% in F1-score). These results indicate that ViMRT is a high-performance method for the extraction of virus mutation from the biomedical literature. Besides, we present a search engine for researchers to quickly find and accurately search virus mutation-related information including virus genes and related diseases.

**Availability and implementation:**

ViMRT software is freely available at http://bmtongji.cn:1225/mutation/index.

## 1 Introduction

Viruses cause many human diseases including cancers by persistent chronic infection and induce public health insecurity (Escandon *et al.*, 2021; Zapatka *et al.*, 2020). Viral mutation is the ultimate source of viral genetic diversity and evolution which plays a key role in the relationship between the virus and disease progression (Sanjuan and Domingo-Calap, 2016). For example, the point mutations of hepatitis B virus (HBV) surface are closely related to immune and vaccine escape, resulting in active viral replication and the development of hepatocellular carcinoma (Zhao *et al.*, 2021). Severe acute respiratory syndrome coronavirus-2 (SARS-CoV-2) variants can cause a very high risk of infection (Araf *et al.*, 2022). Substantial virus research has been prompted by the importance of virus mutation across many diverse clinical and basic research fields and reported in PubMed with constant updates, which provides an invaluable resource for obtaining key information about virus genomic variants. Therefore, it is of great significance for clinical diagnosis and treatment to perform extraction and relationship analysis of virus-related mutations, genes and disease entities by the efficient use of the rich literature.

However, those unstructured texts are limited by the insufficient feasibility of traditional manual curation in utilization, as it cannot be carried out on all of the available literature. Furthermore, due to the inconsistency of virus mutations and the lack of standard mutation nomenclature, the conventional ‘Keyword Search’ may be difficult to quickly and non-redundantly get desired mutation information from literature in PubMed. As such, extracting, mining and integrating virus mutation-related information from those extremely large amounts of literature has become an increasingly important task in many downstream applications, such as virus mutation database development (Davey *et al.*, 2014; Wang *et al.*, 2022), immune escape mechanism exploration (Huang *et al.*, 2020), vaccine design and updating (Xie *et al.*, 2021), drug resistance mutation analysis and assistance in clinical personalized medicine (Chen *et al.*, 2020; Yeo *et al.*, 2020).

With the wide application of text mining and natural language processing in the biomedical field, many named-entity recognition and named-entity normalization tools for mutation have been successively developed to overcome the problem of mutation recognition from the biomedical literature (Lee *et al.*, 2021). For example, MutationFinder uses about 700 regular expressions to identify individual nucleotide or amino acid point mutations or the mutations in simple natural language forms (Caporaso *et al.*, 2007). Extractor of mutation can identify insertions or deletion mutations in addition to point mutations based on regular expression (Doughty *et al.*, 2011). tmVar comprehensively uses conditional random fields (CRF) with regular expression for mutation recognitions (Wei *et al.*, 2013). AVADA is another machine learning and regular expression-based mutation extraction tool which improved gene-mutation mapping in full-text papers (Birgmeier *et al.*, 2020). nala mines natural language processing mutations from the literature (Cejuela *et al.*, 2017). However, those tools aimed at identifying human gene mutation. The accuracy and recall largely reduce when they are applied to identify virus mutation due to the specific written forms of virus mutation significantly differing from human mutation. For example, most written forms often contain slash symbols resulting from occurring multiple mutations at a single site, e.g. E138A/G/K/Q/R/S in human immunodeficiency virus (HIV) reverse transcriptase region (Martin-Alonso *et al.*, 2020). The other popular form of virus variant nomenclature includes the lowercase virus gene at the beginning, e.g. sG145R in the HBV S gene (Chen *et al.*, 2020). Consequently, developing a tool for virus mutation from free text is promising but remains a challenge especially for a variety of writing styles and the shortage of a unified nomenclature as shown by the human genome variation society (HGVS) (den Dunnen *et al.*, 2016). Moreover, extracting the relationship of virus mutation-related entities is also of great biological and clinical application in virus research, especially in genes and diseases (Araf *et al.*, 2022; Soulie *et al.*, 2020). There are several semantic search engines for linking genomic variant data, such as LitVar (Allot *et al.*, 2018), PubTator (Wei *et al.*, 2019), but their information focuses on human mutations or keyword-based search, which hinders quality improvement (sensitivity and specificity) of search results.

To address the above difficulties and challenges, this study developed a novel localization recognition tool of virus mutation to extract and standardize mutation data from available literature, and further integrated the co-occurrence information of virus mutation between viral gene and human disease, with an attempt to design a user-friendly search engine to serve the requirement of researchers to quickly search and download mutation related information, thereby aiding experimental design and clinical guidelines.

## 2 System and methods

**Fig. 1.**
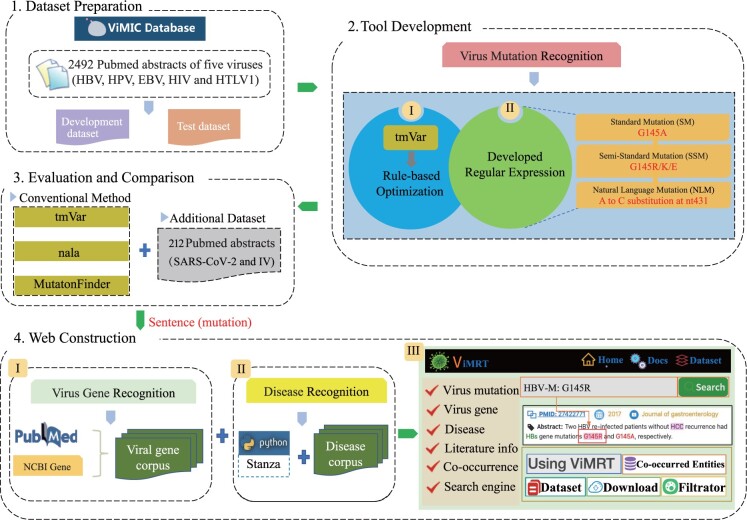
A schematic overview of the ViMRT project. There are four major parts: dataset preparation (development dataset and test dataset construction), virus mutation recognition tool development (rule-based optimization and developed regular expression), evaluation and comparison (conventional method and additional dataset) and web construction (virus gene recognition, disease recognition and web interface design)

An overview of ViMRT is summarized in Figure 1, including virus dataset preparation, mutation recognition tool development, performance evaluation and web construction.

### 2.1 Virus dataset preparation

The gold standard file of virus mutation was collected from our previously developed ViMIC database (Wang *et al.*, 2022), consisting of mutation-related literature in five viruses, HBV, HIV, human papillomavirus (HPV), human T-cell lymphotropic virus type-1 (HTLV1) and Epstein-Barr virus. In this study, the positive samples consisted of 1246 abstracts containing virus mutation information. To balance the positive and negative samples, 1246 abstracts with no virus mutation information were selected as the negative samples. All collected virus literature was divided into a development dataset and a test dataset on a scale of 1:2. In total, 415 positive abstracts and 415 negative abstracts were used as the development dataset, and 831 positive abstracts and 831 negative abstracts were used as the independent test dataset. All virus datasets are available at: http://bmtongji.cn:1225/mutation/dataset.

### 2.2 Mutation recognition tool development

ViMRT has two independent modules to identify mutations as follows:

#### 2.2.1 Module 1: Optimizing the recognition result of tmVar by rule patterns

In this module, we first used the tmVar (Wei *et al.*, 2013) to obtain mutation information and then we built eight rule patterns to correct and standardize mutation identification of tmVar based on the site of mutation in the original sentences and mutation displacement (Table 1). This mainly included two aspects: negative results (identified entities are not actual virus mutations) and positive results (identified entities are actual virus mutations). For negative results, our rules mainly dealt with three recognition errors: multiple mutations linked with a slash (‘A1762T/G1764A’ was identified as ‘A1762T/G’ by tmVar), non-sense mutation (‘Q118R/Stop’ was identified as ‘Q118R/S’ by tmVar) and the three-letter abbreviation of amino acid (‘Pro130Ile/Thr/Ser’ was identified as ‘Pro130Ile/T’ by tmVar). The mentioned errors resulted from incomplete extractions of words. Thus, the recognition result can be optimized by completing words in sentences, and the incorrect recognition part was excluded after optimization. For example, ‘A1762T/G’ by tmVar will be extended into ‘A1762T/G1764A’ and normalized as ‘A1762T’ and ‘G1764A’, without the negative result ‘A1762G’. For positive results, our rules mainly standardized five written forms: mutation with lowercase viral gene (rtM204V), simple natural language written form with a linking preposition (a mutation from T to G at nucleotide 178), mutation written form with ‘>’ (8403A>G), a word beginning with mutation level (p.Lys316Glu) and a special written form of HPV mutant (E-C109G).

**Table 1.** The optimization rules of virus mutation recognized by tmVar

Result type	Rule patterns	Examples
False positive	Include ‘\d{1,}\D{1,}\d{1,}|[/,]’ & no lowercase letters and not end with an ‘S’	A1762T/G1764T (A1762T/G^a^)
	End with an ‘S’ & include ‘STOP|stop|Stop|St’	Q118R/Stop (Q118R/S^a^)
	Include the three characters of 20 amino acids	Pro130Ile/Thr/Ser (Pro130Ile/T^a^)
True positive	Include abbreviation of virus gene	rtM204V (M204V^b^)
	Natural language include ‘to|with|by|[-]|for|ins|del|Non|Delta|of|at’	A mutation from T to G at nucleotide 178 (T178G^b^)
	Include ‘>’ and not include lowercase ‘l|u|s|p|r|y|h|i|n|e|o|m|v’	8403A>G (A8403G^b^)
	Begin with mutation levels (c.|g.|p.)	p.Lys316Glu (K316E^b^)
	Special written form of HPV mutant and include ‘A|C|E|G|T’	E-C109G (C109G^b^)

a The identification errors. For example, A1762T/G1764T are identified A1762T/G; This will be mistaken for A1762T and A1762G in the final identification results.

b The normalized mutation written forms.

#### 2.2.2 Module 2: Developing regular expression patterns to recognize virus mutation

Based on the development dataset and false positive results of tmVar, we classified the written form of virus mutation into three signatures for nucleotide and amino acid mutation: standard mutation (SM: written form of mutation with no punctuation), semi-standard mutation (SSM: written form of mutation containing punctuation) and natural language mutation (NLM: written form of mutation with the natural language) and then constructed corresponding regular expression patterns (Table 2). Finally, we combined the united results of the above two modules as the final recognition result of ViMRT with a standardized written format which consisted of the wild type with uppercase letter, the location number of the mutation and the mutated type with uppercase letter.

**Table 2.** Regular expression patterns of virus mutation based on development dataset

Type	Signatures	Regular expression patterns	Examples
Amino acid	SM	[AC-IK-NP-TV-Y](/[AC-IK-NP-TV-Y]){0,}([1-9]\d{0,3})((STOP|stop|Stop|St\.|\*|[AC-IK-NP-TV-Y]))(/(STOP|stop|Stop|St\.|\*|[AC-IK-NP-TV-Y])){0,}	M204I
		[AC-IK-NP-TV-Y][1-9]\d{1,3} ?(STOP|Stop|stop|St\.|\*)	W196stop
		([AC-IK-NP-TV-Y]|[ACGHILMPSTV][aehilrsy][aeglnoprstuy])(-?[1-9]\d{1,3}-?)([ACGHILMPSTV][aehilrsy][aeglnoprstuy]|Stop)(?![-\d])	C144Lys
	SSM	[ACGHILMPSTV][aehilrsy][aeglnoprstuy](/[ACGHILMPSTV][aehilrsy][aeglnoprstuy]){1,}(-?[1-9]\d{1,3}-?)([ACGHILMPSTV][aehilrsy][aeglnoprstuy]|Stop)(/[ACGHILMPSTV][aehilrsy][aeglnoprstuy]|Stop){1,}	Met204Ile/Val
		([1-9]\d{1,3}-?[AC-IK-NP-TV-Y]|[AC-IK-NP-TV-Y][1-9]\d{1,3})--(&gt;|>)[AC-IK-NP-TV-Y]')	555-V-->I
		(A(la|rg)|As[np]|Cys|Gl[nuy]|His|Ile|L(eu|ys)|Met|P(he|ro)|Ser|T[hy]r|Trp|Val)-[1-9]\\d{1,3}--(&gt;|>) (A(la|rg)|As[np]|Cys|Gl[nuy]|His|Ile|L(eu|ys)|Met|P(he|ro)|Ser|T[hy]r|Trp|Val)	Val-555-->Ile
	NLM	([ACGHILMPSTV][aehilrsy][aeglnoprstuy])(\(?)([1-9]\\d{1,3})(\))([-]to[-]|--(&gt;|>))([ACGHILMPSTV][aehilrsy][aeglnoprstuy])	Leu (526)-to-Met
		([ACGHILMPSTV][aehilrsy][aeglnoprstuy])(?P<site>[1-9]\d{1,3})([-]to[-]|--(&gt;|>))([ACGHILMPSTV][aehilrsy][aeglnoprstuy])(?P=site)	Val184-to-Ala184
Nucleotide	SM	[ACGT](/[ACGT]){0,}([1-9]\d{2,4})[ACGT](/[ACGT]){0,}	G1613A
	SSM	[1-9]\d{2,4}[a-z\( ]*?[ACGT]--(&gt;|>)[ACGT][\)]?	1762 (A-->T)
		[1-9]\d{2,4} \([ACGT]\-[ACGT]\)	1896 (G-A)
	NLM	[ACGT][-]to[-][ACGT] .*?at .*?nt.*?[1-9]\d{2,4}	G-to-A mutation at nucleotide (nt) 1896

*Note*: Classification of mutation signatures as found in the literature.

SM, standard mutation; SSM, semi-standard mutation; NLM, natural language mutation.

### 2.3 Performance evaluation

To evaluate the performance of ViMRT, we compared ViMRT with three other classic tools (tmVar, MutationFinder and nala). Moreover, we used the additional dataset including the abstracts of influenza virus (IV) and SARS-CoV-2 to further evaluate the recognition effect in other viruses. We calculated three different parameters: Precision, Recall and F1-score. The three parameters were defined as follows:

(1) $$\mathrm{Precision}=\;\frac{\mathrm{TP}}{\mathrm{TP}+\mathrm{FP}},\;$$

(2) $$\mathrm{Recall}\;=\;\frac{\mathrm{TP}}{\mathrm{TP}+\mathrm{FN}},\;$$

(3) $$F1-\mathrm{score}\;=\;\frac{2*\mathrm{Precision}*\mathrm{Recall}}{\mathrm{Precision}+\mathrm{Recall}}\;,$$

where TP, FP and FN represent the numbers of true positive (identified entities are actual virus mutation), false positive (identified entities are not actual virus mutation) and false negative (identified non-virus mutation entities are actual virus mutation), respectively.

### 2.4 Web construction

#### 2.4.1 Virus genes extraction

We first built the virus gene corpus from PubMed and the NCBI gene database (https://www.ncbi.nlm.nih.gov/gene/) due to the particularity of different viral genes and then developed Python scripts to identify the virus genes from the sentences including virus mutation.

#### 2.4.2 Virus mutation related diseases annotation

We used the Stanza natural language processing (NLP) library (https://github.com/stanfordnlp/stanza) for many human languages to identify the diseases from the sentences including virus mutation and further developed Python scripts to extract related-disease information based on disease corpus collected from CTD (http://ctdbase.org).

#### 2.4.3 Web search engine development

To quickly search and find the interactions of virus mutation and related information for researchers, we used ViMRT to identify virus mutations, genes and diseases from the 9629 PubMed abstracts and 3160 PMC full-text articles and calculated the co-occurrence correlation of the variant and disease in sentences by the Fisher's test. We finally constructed the user-friendly web pages for visual search and display using Django 3.2.6.

## 3 Results

### 3.1 Virus mutation dataset

**Fig. 2.**
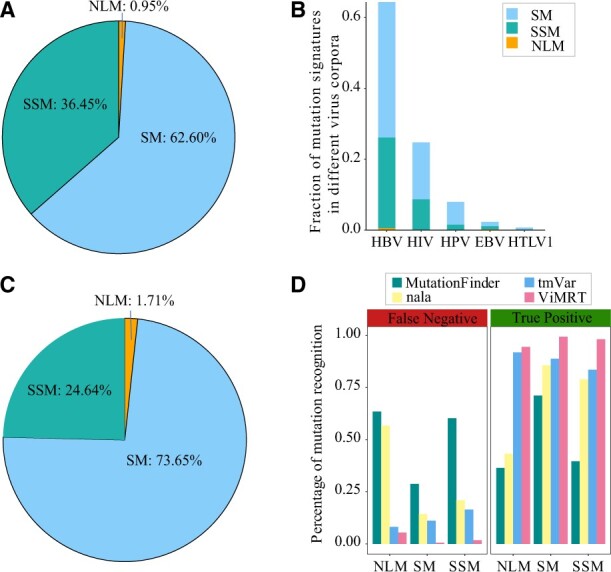
The percentage of different mutation signatures in our corpus and comparison of recognition results. (**A**) The percentage of different mutation signatures in the total corpus (1246 abstracts). (**B**) The percentage of different mutation signatures in the corpus of different viruses. (**C**) The percentage of different mutation signatures in SARS-CoV-2 and IV corpus (212 abstracts). (**D**) The percentage of false negative and true positive mutation signatures in the test dataset by ViMRT, MutationFinder, nala and tmVar, respectively

Viral mutation gold data contained 1246 abstracts on five viruses (2349 mutations) from the ViMIC database. The development dataset and test dataset were allocated in a ratio of 1:2. In total, SM accounted for 62.60% of total annotations. SSM was 36.45% and the fraction of NLM was only 0.95% (Fig. 2A). The fraction of mutation signatures in the corpus of different viruses is shown in Figure 2B.

### 3.2 Evaluation and comparison with existing tools on different datasets

To evaluate the performance of ViMRT, we compared recognition results with three other conventional tools, tmVar, nala and MutationFinder in the test dataset. Notably, Table 3 shows that ViMRT outperformed three other methods with the best two evaluation metrics (Recall: 98.95%, F1-score: 99.17%) and the precision also reached 99.39%. ViMRT displays the state-of-art performance to recognize the mutation of five viruses from the literature used in this study. To further estimate the application of our model in other viral contexts, we manually annotated 212 mutation-related abstracts of SARS-CoV-2 and IV. Figure 2C shows the fraction of mutation signatures. The recognition results are presented in Table 4. The performance of our method was higher than that of the three other methods, which also shows the feasibility and effectiveness of our method.

**Table 3.** Performance evaluation of mutation recognition using different tools on test dataset

Tool	Precision (%)	Recall (%)	F1-score (%)
ViMRT	99.39	**98.95**	**99.17**
tmVar	91.50	69.10	78.74
Nala	93.81	68.80	79.38
MutationFinder	**99.81**	59.26	74.37

The bold values indicate the best value per metric.

**Table 4.** Performance evaluation of mutation recognition using different tools on SARS-CoV-2 and IV dataset

Tools	Precision (%)	Recall (%)	F1-score (%)
ViMRT	99.11	**94.95**	**96.99**
tmVar	96.96	92.72	94.79
nala	97.15	84.59	90.44
MutationFinder	**100**	78.25	87.80

The bold values indicate the best value per metric.

### 3.3 Recognition comparison with existing tools on SM, SSM and NLM

We further compared the recognition results of four methods in three mutation signatures (Table 5). For SM, four methods could perform well in mutations that resembled the HGVS nomenclature. However, ViMRT and tmVar could identify the gene mixed-written form and nonsense mutations that were not identified by nala or MutationFinder. For SSM, ViMRT, tmVar and nala could identify a large proportion of written forms, but only ViMRT could correctly detect SSM which contains more than two slashes. For NLM, ViMRT, tmVar and nala could discern the mutations in simple written form of natural language. Figure 2D shows the percentage of different mutation signatures with true positive results and false negative results by ViMRT, MutationFinder, nala and tmVar. ViMRT performed more accurately than the three other tools, which had the highest percentage of positive results and the lowest percentage of negative results.

**Table 5.** Comparison of different tools on the recognition ability of SM, SSM and NLM

Signatures	Examples	ViMRT	tmVar	nala	Mutation-Finder
*SM*	M204I; Asp8Asn	Yes	Yes	Yes	Yes
	rtI233V	Yes	Yes∗	No	No
	Y225del; 7434CIns	Yes	Yes	Yes	No
	W182 stop; C69∗	Yes	Yes∗	No	No
*SSM*	145G>T; C2288A/T	Yes	Yes	Yes	Yes∗
	L180M+M204V	Yes	Yes	Yes	No
	Y143R/C/D/G; A12S/P33S/P46S	Yes	Yes∗	Yes∗	No
*NLM*	Asn116-to-Thr116	Yes	Yes	Yes	Yes∗
	Alanine 206 had been replaced by a serine	Yes	Yes	Yes	No
	G-A transition at position 5503	Yes	Yes	No	Yes∗

∗The methods can incompletely recognize the examples listed.

SM, standard mutation; SSM, semi-standard mutation; NLM, natural language mutation.

### 3.4 The ViMRT web search engine

**Fig. 3.**
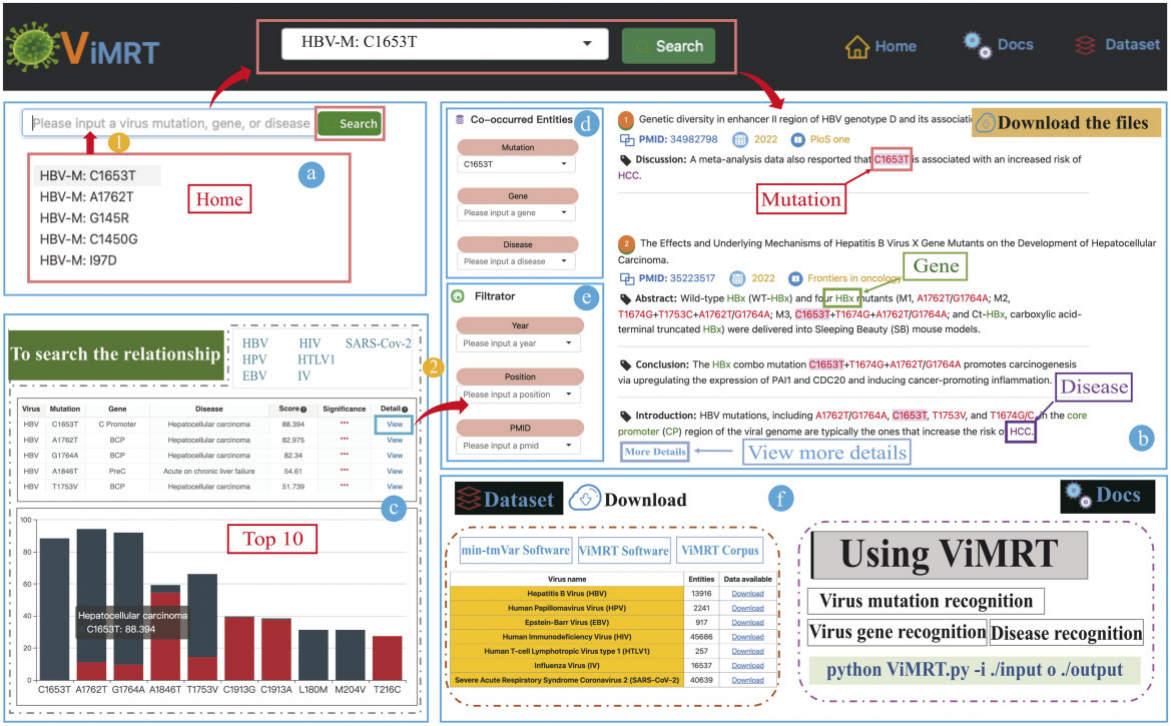
The ViMRT user interface and the usage guidance locally. Multiple clearly delimited zones allow users to perform search and visualize results, which include: (a) A query entity can be inputted on the home page, such as a virus mutation, virus gene or related disease. (b) The match query includes the detailed information of virus mutations, virus genes, and related diseases, sorted by the publication year in a descending order. (c) The co-occurrence correlation of viral mutation and related disease. The table shows co-occurrence score of mutation which have co-occurred in the same sentence with one disease by Fisher's test in each virus (***P < 0.001; **P < 0.01; *P < 0.05; ns, not significant). The histogram chart shows the co-occurrence correlation score of mutation and disease ranking top 10. (d) The user can select the entities menu to get sentence co-occurrence of different entities. (e) Users can also filter publications based on ‘Year’, ‘Position’ and ‘PMID’ of publication matching the query. (f) Users can download mutation data on the ‘Dataset’ page and view the usage of ViMRT in the ‘Docs’ page with a detailed script

We developed the ViMRT web search engine by identifying mutations in 9629 abstracts and 3160 full texts and covered the annotations of viral genes and related diseases. It can be accessed through an easy-to-use graphical web interface, as shown in Figure 3. The web provides two ways to get variant information. One is a simple search. After users enter a query entity in the select bar on the home page, including virus mutation, gene or disease (Fig. 3a), the web will return the best match entity results in its database (Fig. 3b), which includes a list of publications containing highlighted entities, sorted by publication date. Another is co-occurrence correlation rank to search if the aim of users is not very clear. Users can firstly click ‘To search the relationship’ button on the home page, which will return co-occurrence correlation of mutation and disease including seven viruses by the histogram chart and table, and click ‘View’ to get detailed information (Fig. 3c). Those processes have three main features:

First, for each result, it returns the searched entity with a red background as well as other entities with different colors including diseases, genes and other mutations (Fig. 3b), which can very efficiently catch the entity information and research work. Second, users can filter the other co-occurrence entities by the table and the filter box at the left (Fig. 3c and d) which are useful to find potential relations (e.g. C1653T mutation of HBV HBx/core promoter region is strongly associated with an increased risk of hepatocellular carcinoma and reported in many literatures). Third, users can also filter literature information through ‘Year’, ‘Position’ and ‘PMID’ of publication (Fig. 3e). The ‘Dataset’ page and ‘Docs’ page of the web provide the download to easily get complete mutation information of seven viruses and the local usage of ViMRT with a detailed script, respectively (Fig. 3f).

### 3.5 The localization usage of ViMRT

The usage of ViMRT can realize three recognitions: virus mutation recognition, virus gene recognition and disease recognition. Firstly, virus mutation recognition includes downloading literature, optimizing the recognition results of tmVar by rule patterns and recognizing virus mutation by regular expression patterns. Secondly, the virus gene is identified by virus gene vocabulary and a gene match program. Thirdly, disease recognition is based on Stanza 1.4.0 and a normalizing script written by Python (v3.8.5). Full usage of ViMRT is minutely displayed on the ‘Docs’ page: http://bmtongji.cn:1225/mutation/Docs.

## 4 Discussion

This study is the first to gather abundant virus mutation information and develop ViMRT to identify virus mutation by precise rules and regular expressions. It has achieved a higher performance for virus mutation recognition than other conventional tools. At the same time, we are the first to develop a search engine web about a variety of viruses for researchers to quickly search mutation-specific information that includes virus gene and related diseases as reported in the biomedical literatures.

ViMRT displays some notable advantages in identifying virus mutation recognition. Firstly, ViMRT curated and created virus mutation information from five viruses closely related to human disease from the literature to comprehensively cover the majority of written forms. Next, based on that information, we designed rule patterns to optimize recognition results of tmVar, and further summarized three mutation signatures termed SM, SSM, NLM which characterized the written form preference of virus mutation in different literatures. Then we developed regular expressions to recognize mutations for each mutation signatures (Table 2). As we have observed, ViMRT showed state-of-art performance compared to three other conventional tools (Table 3), especially for the written form of virus mutation containing punctuation or gene. We also developed a specific search engine for seven viruses to realize mutation information visualization and mined the buried co-occurrence relationships with related diseases in the original paper, which can contribute to more quickly and accurately retrieving evidence aiding both basic research and clinical application compared to LitVar and Pubtator.

In addition, we observed that ViMRT showed a slightly decreased but still good performance (all indicators > 0.9) to recognize the mutation of SARS-CoV-2 and IV compared to five viruses (Table 4), while the performance of three other tools on the corpus of SARS-CoV-2 and IV were better than those in the test dataset. The possible interpretation is that a lower proportion of SSM in SARS-CoV-2 and IV corpus caused the rise of recall of three other tools (Fig. 2C). Also, mutation of different viruses in the datasets existed in some particular written forms, such as nsp6-L3606fs and spike-glycoprotein-V6fs variants of SARS-CoV-2 (Zekri *et al.*, 2021). Furthermore, we analyzed false positive results of ViMRT, which was mainly caused by terms that are highly similar to the written form of virus mutation, such as human hepatoma cells (C3A) (Nishizawa *et al.*, 2016), where C3A was misidentified as a mutation.

Several future directions can extend this work. Firstly, future study is warranted to expand the rules and regular expressions for more viruses with the emergence of special written forms. With the rapid growth of literature data, we will continue to expand our corpus of viruses, add more virus types and regularly update the literature of our search engine. Secondly, due to the particularity of writing styles of virus mutations, the current version of ViMRT mainly used the rule-based NLP method. In a preliminary study, we have explored whether it is possible to perform a binary classification task using machine learning models for discriminating sentences containing the mutation information to reduce the false positive. Both F1-scores of machine learning methods based on TF-IDF word embedding and TextCNN model could reach 0.8. In the future, with continuous updates and expansion of the virus data set constructed in this study, the state-of-the-art pre-trained language models, such as BERT (Devlin *et al.*, 2019), BioBERT (Lee *et al.*, 2020), PubMedBERT (Gu *et al.*, 2020), will be worth attempting. Thirdly, we will continue to extract and update more buried biological relationships associated with a viral function such as receptor binding and drug resistance.

## Funding

This work was supported by the National Natural Science Foundation of China [81972914 to X.Y.Z. and 81573023 to X.Y.Z.]; the Fundamental Research Funds for the Central Universities [22120200014 to X.Y.Z.]; and Shanghai ‘Rising Stars of Medical Talent’ Youth Development Program [2019-72 to Y.W.].

*Conflict of Interest*: none declared.

## Data Availability

ViMRT software and the web are freely available at: http://bmtongji.cn:1225/mutation/index.
